# The superiority of manual over automated methods in identifying bronchial trees on identical CT images

**DOI:** 10.1038/s41598-022-09401-8

**Published:** 2022-03-30

**Authors:** So Takata, Kotaro Miyake, Atsushi Kumanogoh

**Affiliations:** 1 Department of Respiratory Medicine and Clinical Immunology, Graduate School of Medicine, Osaka University, 2-2 Yamadaoka, Suita, Osaka 565-0871 Japan; 2 Department of Immunopathology, World Premier International Research Center Initiative, Immunology Frontier Research Center, Osaka University, Suita, Osaka Japan; 3 Integrated Frontier Research for Medical Science Division, Institute for Open and Transdisciplinary Research Initiatives, Osaka University, Suita, Osaka Japan; 4 Center for Infectious Diseases for Education and Research, Osaka University, Suita, Osaka Japan

**Keywords:** Image processing, Software

## Abstract

The purpose of this study was to compare a manual bronchoscopic navigation technique, the direct oblique method (DOM), with conventional virtual bronchoscopic navigation software in terms of bronchial identification ability involving reconstruction of a whole bronchial tree from identical CT images. A whole bronchial tree was drawn using manual bronchial recognition with the DOM. The tree was compared with that reconstructed by SYNAPSE VINCENT bronchoscopic navigation-dedicated software. The number of bronchial generations at each terminal tip was then compared between the two approaches. Physicians spent 20 h tracing all bronchi on CT scan images and obtained a bronchial tree. The hand-made bronchial tree had five times the number of tips as that reconstructed by automatic bronchial recognition (1482 vs. 279 tips, respectively). The number of bronchial generations prior to each terminal tip was larger with the DOM than with VINCENT (median, 10; interquartile range (IQR), 9–11 vs. median, 5; IQR, 5–7, respectively; p-value < 0.001). Using the CT image data in this case, manual bronchial recognition with the DOM identified more bronchi than automatic bronchial recognition. This result implies that manual bronchial recognition is a valid basis for detailed bronchoscopic navigation analysis.

## Introduction

Bronchial segmentation on CT images is fundamental to respiratory medicine, including navigated bronchoscopy and the diagnosis of airway diseases. Although software-based automatic bronchial identification is improving, it still cannot identify all bronchi on CT images^1^. Recently, we developed the direct oblique method (DOM), a manual but ultra-precise bronchoscopic navigation technique. However, it remains unclear how the DOM and conventional virtual bronchoscopic navigation compare regarding their bronchial identification ability.

## Methods

A full-chest, thin-slice CT scan (resolution, 512 × 512; slice thickness, 0.625 mm; and slice distance, 0.625 mm) of a patient who underwent chest screening in Osaka University Hospital was analyzed. The whole bronchial tree was manually drawn by direct bronchial recognition on CT images and visualized on a Ziostation2 CT bronchoscopy navigation system (referred to as “Ziostation2”; Ziosoft, Tokyo, Japan). All bronchi and bifurcations were handwritten one by one throughout the lungs. Transverse and longitudinal cross-sectional oblique CT images were used to identify each of the bronchi, just as with the DOM. This procedure is shown in the Supplementary Video. The resultant bronchial tree was compared with that derived using automatic bronchial recognition by SYNAPSE VINCENT bronchoscopic navigation-dedicated software (referred to as “VINCENT-BFsim”; Fujifilm Medical, Tokyo, Japan).

To quantify bronchial identification ability, the number of bronchial generations at each terminal tip was counted. We referred to segmental bronchi as the second generation, subsegmental bronchi as the third generation, and so forth.

The number of bronchial generations prior to each terminal tip was compared with Student’s t-test using R ver. 4.0.2 software (available at http://www.R-project.org; R Foundation for Statistical Computing, Vienna, Austria). A p-value of < 0.05 was considered statistically significant.

This study was approved as Protocol #864 by the Research Ethics Committee of Osaka University. The research was performed in accordance with the Declaration of Helsinki. Written informed consent was obtained from the patient whose CT scan images were used in this study.

## Results

**Figure 1:**
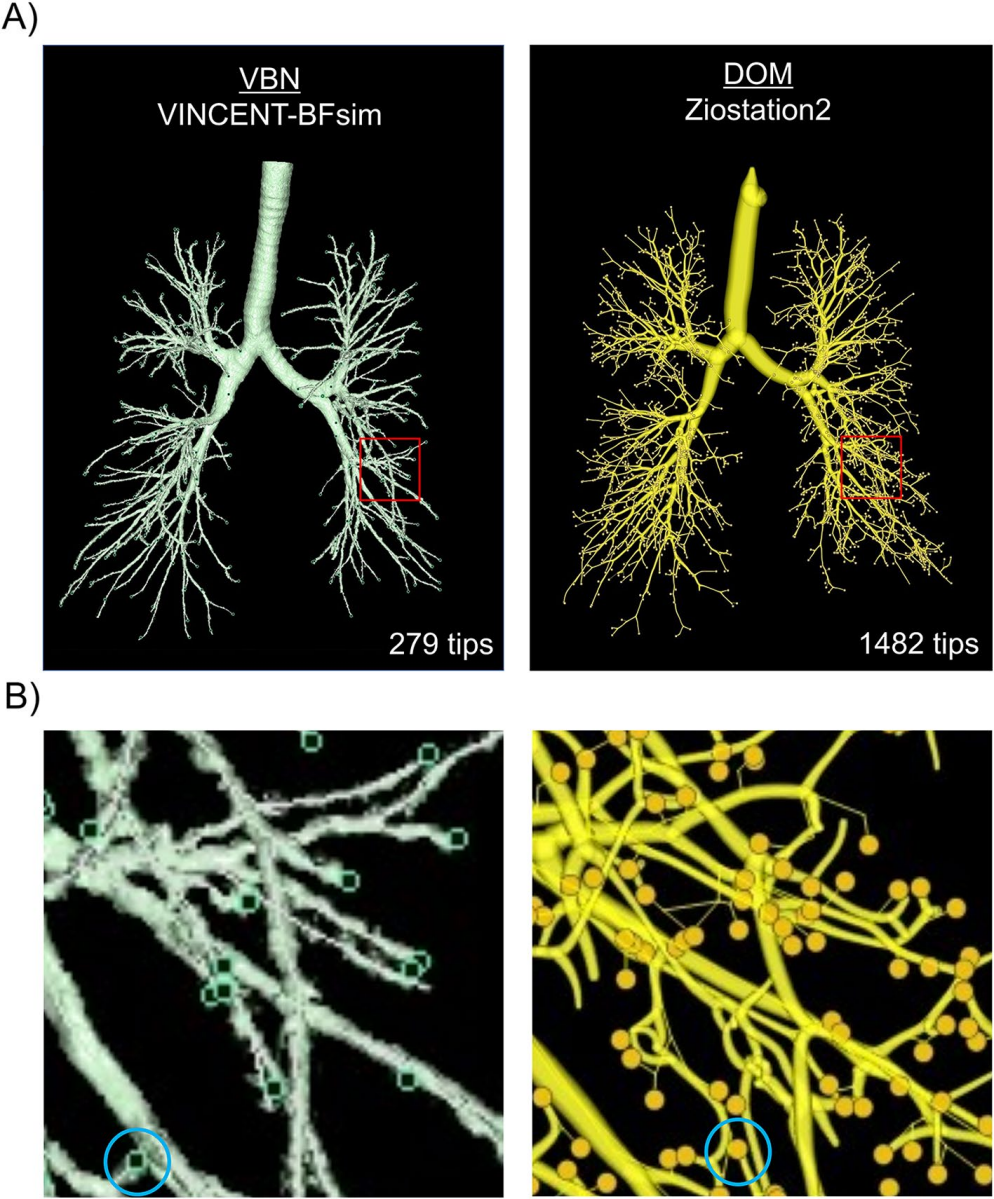
Bronchial trees were reconstructed by automatic CT analysis using virtual bronchoscopic navigation software and by manual CT analysis using the direct oblique method (DOM). (**A**) Images of the entire bronchial trees. The virtual bronchoscopic navigation software, VINCENT-BFsim, identified 279 bronchial tree tips in the whole lung, while the DOM on Ziosation2 identified 1482 tips. The areas indicated by red squares are magnified in (**B**). (**B**) Magnified images of the bronchial trees. The tips of the bronchial trees are shown as round dots. The blue circles indicate examples of tips.

**Figure 2:**
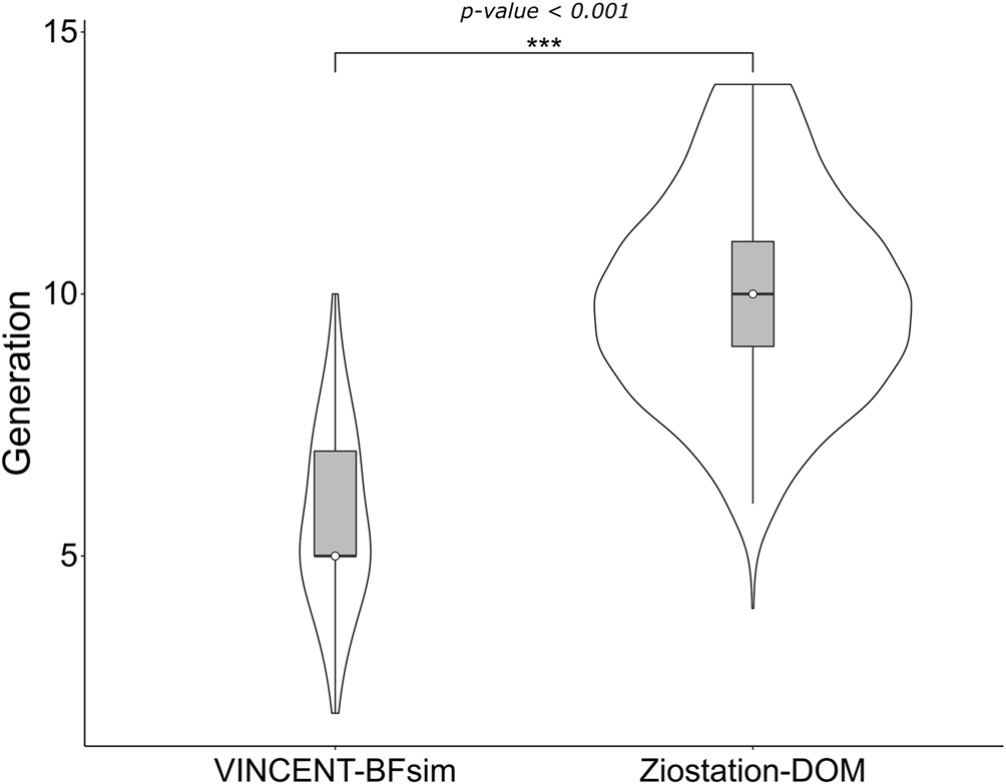
Comparison of the number of bronchial generations prior to each terminal tip. Analysis was performed using Student’s t-test. Triple asterisk means that the p-value is less than 0.001.

**Figure 3:**
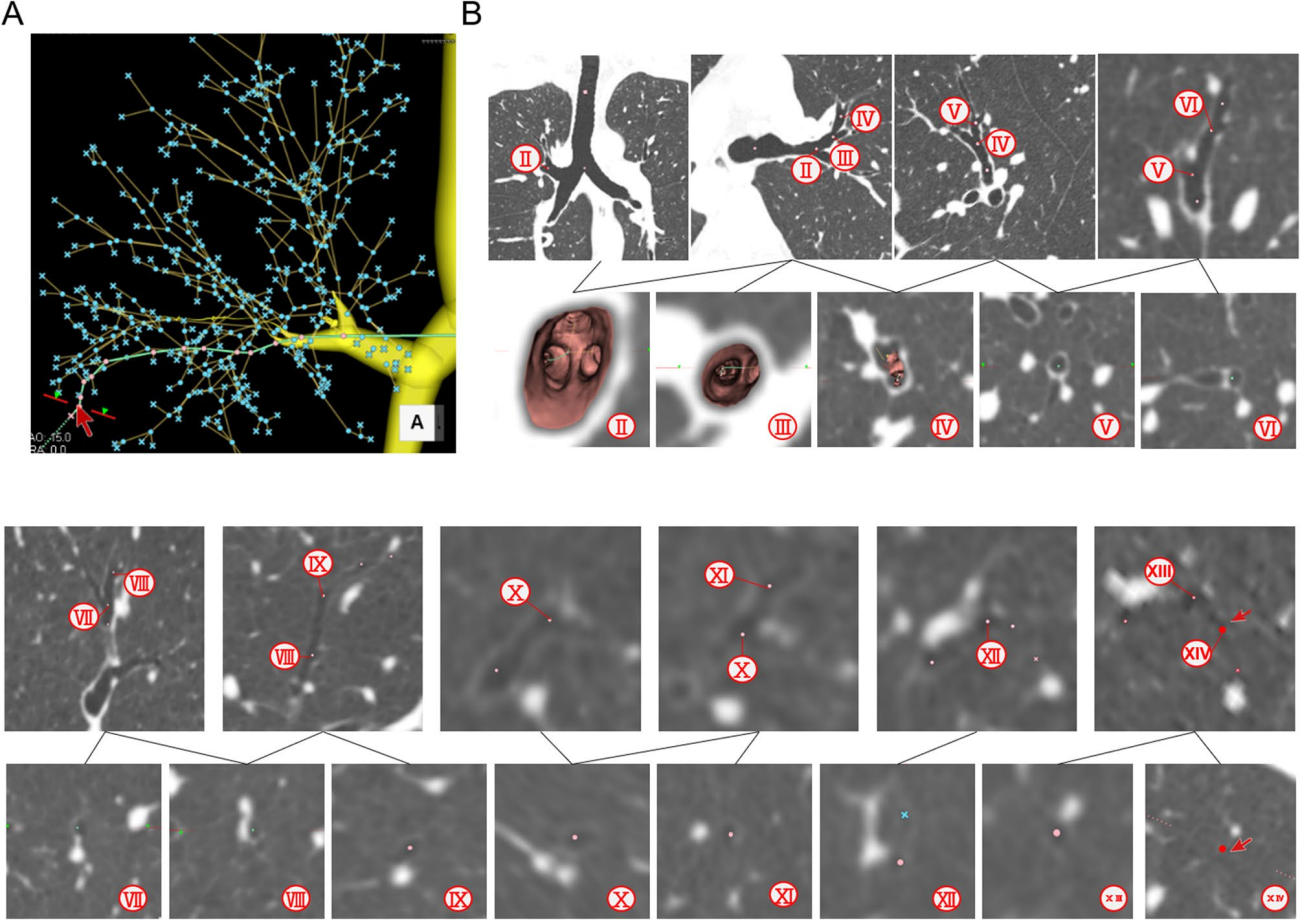
The bifurcations of a 14-generation bronchus detected by Ziostaion2 using the DOM. (**A**) On the bronchial tree pathway indicated by the green line, the pink dots represent bifurcation points and the pink X indicates the tip of the bronchus detected by the DOM. (**B**) Longitudinal cross-sections (upper panel) and transverse cross-sections (lower panel) of the bronchus. The circled numbers indicate the bronchial generation. We used Ziostation2 software to analyze virtual bronchoscopic images of the second- to fourth-generation bronchi, and switched to multi-planar reconstruction to manually detect bronchi of the fifth generation and higher. The transverse cross-sectional images were obscure after about the seventh-generation bronchi. However, it was still possible to obtain information on bronchial bifurcation, since when we placed the cursor (red arrow) on another bronchus in the longitudinal cross-section image, the cursor appeared simultaneously on the transverse cross-sectional image as a virtual bronchoscopy–like dimension. For instance, on the transverse cross-sectional image of the 14th-generation bifurcation, bifurcations are located in the directions of 10 o’clock (red dot) and 4 o’clock (red arrow).

Physicians spent 20 h tracing all bronchi in CT scan images to obtain a bronchial tree. On the other hand, VINCENT-BFsim extracted the bronchial tree in less than 2 min. The bronchial tree identified by the DOM on Ziostation2 had five times the number of tips as that depicted by VINCENT-BFsim (1482 vs. 279 tips, respectively) (Fig. 1). The number of bronchial generations prior to each terminal tip was significantly greater with the DOM than with VINCENT-BSsim (median, 10; interquartile range: IQR, 9–11 vs. median, 5; IQR, 5–7, respectively; p-value < 0.001) (Fig. 2), while the maximum number was 14 for the DOM (Fig. 3) and 10 for VINCENT-BFsim. These results demonstrated that the DOM identified more bronchi than the automatic system.

## Discussion

This study demonstrated that manual handwriting of bronchi on CT images remains a valuable approach today, since many bronchi were not identified by a current software-based automatic bronchial identification method. To quantify the performance of bronchial identification, we compared the number of tips and bronchial generations prior to each terminal tip of the bronchial tree. Although only one bronchial tree was drawn due to the strenuous work involved, the DOM restored the bronchial tree more accurately than the automatic method in this case.

There have been significant advances in CT-based bronchial segmentation methods and bronchoscopic navigation. Conventional manual navigation techniques sometimes yield incorrect and insufficient information because they are based on bronchial recognition on axial CT-images with mental reconstruction of three-dimensional structures^2^. Bronchoscopic navigation systems with automatic bronchial segmentation improve diagnostic yield^3–7^, but their ability to recognize bronchi is still inadequate and varies between software systems. For example, when compared to LungPoint (Broncus Medical, Mountain View, CA), a globally used bronchoscopic navigation system, VINCENT-BFsim has better bronchial segmentation performance^1,8^ but it still cannot identify all bronchi on CT images near the target lesion^1^. The DOM is a manual navigation technique, which makes it possible to use all bronchi on CT images for bronchoscopic navigation^1^. However, unlike in conventional manual analysis, the DOM does not require mental reconstruction because oblique sections are used instead of axial images. In addition, bronchi are carefully evaluated in both the transverse and longitudinal cross-sections of each bronchus^1^. Since a limited area of analysis is needed for bronchoscopy preparation, the DOM takes only about 5 min per case^1^, which is almost equivalent to VINCENT-BFsim.

We propose the efficacy of bronchoscopic navigation systems such as Ziostation2 in which automatic bronchial identification and precise handwriting are seamlessly fused. Further study is needed to determine whether such precise navigation improves diagnostic rates.

## Supplementary Information

Supplementary Video Legend.

Supplementary Video 1.
