# Supplementary material for: The superiority of manual over automated methods in identifying bronchial trees on identical CT images
Source: Sci Rep. 2022 Mar 30;12:5416. doi: 10.1038/s41598-022-09401-8 (PMC8969159; doi:10.1038/s41598-022-09401-8)
Supplement: Supplementary file 1 — Supplementary Video Legend. [file 41598_2022_9401_MOESM1_ESM.docx]

Supplementary Video.

**Manual identification of bronchi using oblique CT images.** In this video, three branches are manually added. Each bifurcation is evaluated in the transverse (left) and longitudinal sections (right). The angle of the longitudinal section is defined by the red and white lines on the transverse section. On the longitudinal section, the location of the transverse section is indicated by red lines.
